# INVESTIGATING CAUSAL LINK BETWEEN VOLUNTEERING AND BLOOD PRESSURE BY GENDER, RACE, AND COHORT

**DOI:** 10.1093/geroni/igae098.2888

**Published:** 2024-12-31

**Authors:** Seoyoun Kim, Cal Halvorsen, Koichiro Shiba

**Affiliations:** Texas State University, San Marcos, Texas, United States; Washington University in St. Louis, St. Louis, Missouri, United States; Boston University, Boston, Massachusetts, United States

## Abstract

Given that nearly half of adults 50 and older have hypertension in the United States, public health interventions focus on reducing risk factors. However, population-based approaches that aim to enhance protective factors, such as social engagement, can be applied to larger groups, resulting in a downward shift in the population blood pressure distribution. Further, understanding the potential heterogeneous effects of volunteering frequency by demographic factors help identify the population subgroups in which volunteering is more (or less) protective against hypertension. The current project examines the link between volunteering and changes in systolic and diastolic blood pressure by gender, race/ethnicity, and cohort as potential effect modifiers. Using the Health and Retirement Study (2006-2016, N=18,847), this study assessed the longitudinal relationships between volunteering (2010/2012) and blood pressures (2014/2016) within each stratum of gender, race/ethnicity, and age cohort. The analyses adjusted for pre-volunteering covariates (2006/2008) as well as selection into volunteering using Inverse Probability Treatment Weighting (IPTW). The joint exposure of volunteering and effect modifiers was estimated by the relative excess risk due to interaction (RERI). Results show that individuals volunteering 201+ hours annually had a lower likelihood of clinically high diastolic (PR=0.84, p=0.04) and systolic (PR=0.98, p=0.04) blood pressure. Moderate level of volunteering was predictive of low systolic blood pressure. The combination of volunteering 201+ hours and being female was protective against high diastolic blood pressure (RERI=-0.09, p=0.02). Given the modest effect modification, frequent volunteering has an anti-hypertensive effect for the general population of adults 50 and older.

